# Physical activity and cancer biology: a narrative review of molecular mechanisms and introduction of the SCRUM-MONSTAR LIFELOG study

**DOI:** 10.1007/s10147-025-02798-y

**Published:** 2025-06-10

**Authors:** Shugo Yajima, Shin Kobayashi, Tadayoshi Hashimoto, Yoshiaki Nakamura, Riu Yamashita, Toshihiro Misumi, Yasutoshi Sakamoto, Satoshi Horasawa, Takao Fujisawa, Mitsuho Imai, Taro Shibuki, Yuichiro Tsukada, Hideaki Bando, Hitoshi Masuda, Takayuki Yoshino

**Affiliations:** 1 Department of Urology, National Cancer Center Hospital East, Kashiwa, Japan; 2 Department of Hepatobiliary and Pancreatic Surgery, National Cancer Center Hospital East, 6-5-1 Kashiwa-no-ha, Kashiwa, 277-8577 Japan; 3 Translational Research Support Office, National Cancer Center Hospital East, Kashiwa, Japan; 4 Department of Gastroenterology and Gastrointestinal Oncology, National Cancer Center Hospital East, Kashiwa, Japan; 5 Division of Translational Informatics, Exploratory Oncology Research and Clinical Trial Center, National Cancer Center Hospital East, Kashiwa, Japan; 6 Department of Data Science, National Cancer Center Hospital East, Kashiwa, Japan; 7 Department of Head and Neck Medical Oncology, National Cancer Center Hospital East, Kashiwa, Japan; 8 Department of Colorectal Surgery, National Cancer Center Hospital East, Kashiwa, Japan

**Keywords:** Physical activity, Molecular residual disease, Cancer, Wearable device, Whole-genome sequencing, Circulating tumor DNA

## Abstract

**Background:**

Physical activity (PA) has been consistently associated with improved cancer outcomes across multiple epidemiological studies. While the evidence for clinical benefits is strong, the underlying molecular mechanisms remain poorly understood. Recent technological advances now enable both continuous monitoring of PA through wearable devices and comprehensive molecular profiling through multi-omics approaches, including whole-genome sequencing (WGS)-based molecular residual disease (MRD) detection. This review examines current evidence regarding PA’s effects on cancer biology and introduces the LIFELOG study, which aims to address critical knowledge gaps in this field.

**Methods:**

We review the current literature on PA and cancer with emphasis on molecular mechanisms, and present the design of the LIFELOG study, an ancillary study to MONSTAR-SCREEN-3. The LIFELOG study will enroll 170 post-surgical cancer patients who will wear the mSafety™ wrist device for continuous PA monitoring. We will investigate associations between PA metrics and multi-omics profiles including WGS-based MRD detection, transcriptome analyses, plasma proteomics, and gut microbiome analyses. The feasibility phase has already begun with encouraging preliminary results regarding device compliance and data quality.

**Discussion:**

Despite substantial evidence supporting PA’s benefits in cancer prevention and survivorship, understanding which specific PA characteristics most effectively influence cancer outcomes remains unclear. The LIFELOG study represents the first comprehensive analysis integrating continuous PA monitoring with molecular profiling in cancer patients. By examining relationships between PA patterns and both MRD dynamics and multi-omics profiles, we aim to identify molecular mechanisms underlying exercise benefits and potentially guide development of evidence-based, precision PA interventions for cancer survivorship.

**Trial Registration:**

This ancillary study (Institutional Review Board number: 2024-111, approved on November 18, 2024) is conducted under the MONSTAR-SCREEN-3 trial platform, which is registered in the UMIN Clinical Trials Registry (UMIN000053975, registered on March 27, 2024).

## Introduction

### Physical activity and cancer outcomes: epidemiological evidence

Extensive epidemiological research has established physical activity (PA) as a key modifiable factor influencing cancer outcomes. A growing body of evidence from observational studies and randomized controlled trials has demonstrated associations between PA and reduced cancer incidence, improved treatment response, and enhanced survival across multiple cancer types [1–4]. Recent studies have shown that even brief (1–2 min) vigorous intermittent lifestyle physical activity (VILPA) can significantly reduce cancer-related mortality [1]. These findings highlight the potential for accessible, non-structured forms of PA to confer substantial health benefits.

The impact of PA appears to vary by cancer type, with particularly strong evidence for cancers of colorectal cancer, renal cell carcinoma, urothelial carcinoma, endometrial cancer, or gastric cancer (cardia) [2]. Moreover, the timing, duration, intensity, and modality of PA may differentially affect cancer outcomes, though the optimal “dose” and characteristics remain unclear. This knowledge gap underscores the need for more precise measurement and characterization of PA in cancer research. In this narrative review, we examine the biological mechanisms through which PA may influence cancer outcomes, with particular focus on immune function and inflammatory processes. The literature search strategy employed for this narrative review is detailed in the Supplementary Appendix. The SCRUM-MONSTAR LIFELOG study, introduced later, represents an innovative approach to address current knowledge gaps by integrating continuous PA monitoring with comprehensive molecular profiling in cancer patients.

### Biological mechanisms underlying exercise effects in cancer

Regular physical activity is increasingly recognized as a crucial factor in oncology, influencing not only cancer prevention but also the prognosis and quality of life (QOL) of patients diagnosed with cancer [1–3]. The biological mechanisms underpinning these benefits are multifaceted, involving systemic and local effects that can modulate tumor development, progression, and response to therapy [4–8]. This section will delve into these mechanisms, expanding on previous understandings by incorporating recent insights from basic and preclinical research, particularly concerning the interplay between exercise, the tumor microenvironment, systemic inflammation, and anti-tumor immunity. A comprehensive summary of these mechanisms and their clinical implications is presented in Table 1.

**Table 1:** Physical activity effects on cancer: mechanisms and clinical implications

Domain	Biological mechanisms	Clinical implications	Key references
Cancer prevention	Modulation of sex hormones (estrogen, testosterone) Regulation of metabolic hormones (insulin, IGF) Reduction of chronic inflammation Improved DNA repair mechanisms Reduction of oxidative stress Maintenance of healthy body weight and adipokine balance	Reduced incidence of multiple cancer types Dose–response relationship with increasing activity levels Protective effects may vary by cancer type	[2, 9–14]
Direct anti-tumor effects	Exercise-induced myokines (irisin, SPARC, oncostatin M) Enhanced NK cell mobilization and tumor infiltration Increased CD8 + T cell recruitment Shift in macrophage polarization (M2 → M1) Modification of tumor microenvironment Reduction of tumor hypoxia	Slower tumor growth Reduced metastatic spread Enhanced immune surveillance Altered tumor behavior and aggressiveness	[7, 8, 15, 16, 20]
Enhanced treatment efficacy	Surgery: Improved cardiovascular fitness Enhanced pulmonary function Chemotherapy/endocrine therapy: Improved tumor vascularization and drug delivery Reduced hypoxia and drug resistance Enhanced immune cell function Radiation therapy: Improved tumor oxygenation Enhanced radiosensitivity Immunotherapy: Increased T-cell priming and activation Enhanced immune infiltration	Surgery: Reduced postoperative complications Faster recovery Shorter hospital stay Chemotherapy/endocrine therapy: Improved treatment tolerance Reduced toxicity Enhanced therapeutic efficacy Radiation therapy: Better tumor control Reduced normal tissue toxicity Immunotherapy: Increased response rates Potential for enhanced durability of response	[7, 17–22]
Quality of life enhancement	Regulation of inflammatory cytokines Neuroendocrine modulation Maintenance of muscle mass and function Circadian rhythm regulation	Reduced cancer-related fatigue Improved physical functioning Better psychological well-being Reduced anxiety and depression Enhanced sleep quality Higher overall QOL scores	[3, 4, 23, 24]

*AMPK* AMP-activated protein kinase, *BDNF* brain-derived neurotrophic factor, *CD8* + cluster of differentiation 8 positive, *CRP* C-reactive protein, *HPA* hypothalamic–pituitary–adrenal, *IGF* insulin-like growth factor, *IL-1β* Interleukin 1 beta, *IL-6* Interleukin 6, *M1/M2* macrophage polarization states, *NF-κB* nuclear factor kappa B, *NK* natural killer, *PGC-1α* peroxisome proliferator-activated receptor gamma coactivator 1-alpha, *QOL* quality of life, *ROS* reactive oxygen species, *SPARC* secreted protein acidic and rich in cysteine, *TNF-α* tumor necrosis factor alpha

#### Cancer prevention

Epidemiological studies have consistently shown an inverse relationship between physical activity levels and the risk of developing several types of cancer, including colon, breast, and endometrial cancers [2, 9]. The proposed mechanisms are diverse. Exercise can modulate sex hormones, such as estrogen and testosterone, and metabolic hormones, including insulin and insulin-like growth factors (IGFs), which are known to play roles in carcinogenesis [10, 11]. Furthermore, regular physical activity plays a crucial role in mitigating chronic inflammation, a key promoter of carcinogenesis. This anti-inflammatory effect stems from its contribution to maintaining a healthy body weight, thereby reducing obesity-related inflammatory states and adipokine dysregulation [12], as well as from the direct capacity of exercise training to lower systemic levels of pro-inflammatory mediators [13]. Emerging research also suggests that exercise can positively influence DNA repair mechanisms and reduce oxidative stress, further contributing to its cancer-preventive effects [14].

#### Prolonging survival in cancer patients

For individuals diagnosed with cancer, physical activity has been associated with prolonged survival across various cancer types. These benefits stem from both direct effects on the tumor and indirect effects via modulation of the host’s physiological environment and enhancement of treatment efficacy.

- **Direct effects on cancer:** Preclinical models have demonstrated that exercise can directly impact tumor growth and metastasis. Exercise-induced factor (e.g., irisin, SPARC, oncostatin M), released from contracting skeletal muscles, can exert anti-tumor effects by inhibiting cancer cell proliferation, inducing apoptosis, and reducing angiogenesis within the tumor microenvironment [15]. For example, exercise can increase the trafficking and infiltration of immune cells, such as natural killer (NK) into the tumor, thereby enhancing immune-mediated tumor cell killing [8, 16].
- **Enhancing treatment efficacy and suppressing complications:** Physical activity can play a significant role in improving the efficacy of conventional cancer treatments, including surgery, chemotherapy, and radiation therapy, while also mitigating their associated side effects.
- **Surgery:** Prehabilitation, or exercise intervention before surgery, can improve functional capacity and reduce postoperative complications, leading to faster recovery and potentially better long-term outcomes [17, 18].
- **Chemotherapy and endocrine therapy:** Exercise has been shown to alleviate common side effects of chemotherapy, such as cancer-related fatigue, neuropathy, and cardiotoxicity [19]. Beyond mitigating side effects, preclinical evidence strongly suggests that exercise can directly enhance the efficacy of cancer treatments. For instance, a systematic review of rodent models demonstrated that exercise significantly improved the effectiveness of various chemotherapeutic agents and the endocrine therapy tamoxifen, often in an additive or synergistic manner [7]. Mechanistically, exercise may achieve this by improving tumor perfusion, reducing hypoxia, and potentially sensitizing cancer cells to cytotoxic and hormonal agents [7, 20].
- **Radiation therapy:** Similar to chemotherapy, exercise can reduce fatigue and improve QOL in patients undergoing radiation therapy. There is also emerging preclinical evidence that exercise might enhance radiosensitivity in tumors [21].
- **Immune checkpoint inhibitors:** The immunomodulatory effects of exercise are garnering considerable attention, and its combination with immune checkpoint inhibitors (ICIs) represents an active and promising area of research for enhancing therapeutic efficacy. Indeed, physical activity can promote the mobilization, cytotoxicity, differentiation, and migration of immune cells, positioning it as a viable strategy to bolster the effectiveness of immunotherapy in cancer patients [22]. Currently, clinical trials, such as NCT05358938, are underway to validate these combined effects of exercise and ICIs in human subjects. Furthermore, the exercise-induced attenuation of chronic inflammation may also contribute to a more favorable tumor microenvironment, thereby potentially increasing the efficacy of ICIs [22].

#### Improving quality of life (QOL)

Beyond survival benefits, physical activity significantly contributes to improving the QOL of cancer patients and survivors. It is effective in managing a wide range of cancer-related and treatment-related symptoms, including fatigue (the most common and distressing symptom), anxiety, depression, sleep disturbances, and declines in physical functioning [23]. Besides, the psychological benefits, such as reduced stress and improved mood, are also crucial components of enhanced QOL [24].

### Advances in cancer monitoring and molecular profiling

Recent technological advances have revolutionized cancer monitoring and characterization. Circulating tumor DNA (ctDNA) analysis now enables highly sensitive detection of molecular residual disease (MRD), allowing for early identification of cancer recurrence and real-time monitoring of treatment response. Whole-genome sequencing (WGS)-based analysis of MRD is expected to provide unprecedented insight into disease status and recurrence risk [25, 26].

Multi-omics approaches, including genomics, transcriptomics, proteomics, and metabolomics, provide comprehensive molecular characterization of tumors and host responses. The SCRUM-Japan MONSTAR-SCREEN-3 trial, a Japanese nationwide cancer genomics initiative, leverages these advances along with spatial transcriptomics and proteomics analyses to comprehensively characterize tumor biology and host responses [27, 28].

### Wearable technology for physical activity monitoring

Traditional assessment of PA in research settings has relied heavily on self-reported questionnaires, which are subject to recall bias and limited in their ability to capture the nuances of PA patterns. The emergence of consumer-grade and research-grade wearable devices has transformed the ability to objectively measure PA in free-living conditions.

The mSafety™ wrist device (Sony Network Communications Inc., Tokyo, Japan) facilitates continuous and objective monitoring of PA in real-world settings [29]. Equipped with multiple sensors, including a configurable inertial measurement unit with an adjustable three-axis accelerometer and gyroscope, and a photoplethysmography (PPG) sensor for pulse wave monitoring, the device offers comprehensive data acquisition. A key advantage of the mSafety™ is its ability to transmit raw data directly to secure cloud servers via LTE-M communication. This capability to access and analyze raw sensor data, rather than pre-processed or interpreted metrics, is a critical strength for researchers seeking a detailed understanding of movement dynamics.

### Knowledge gap and study rationale

Despite these advances in both molecular analysis and PA monitoring, no study has examined the relationship between PA and molecular profiles in cancer patients. Understanding this relationship could provide crucial insights into the mechanisms through which PA influences cancer outcomes and potentially inform the development of evidence-based PA interventions.

As precision medicine evolves to incorporate both molecular profiling and lifestyle factors, there is a critical need to understand how PA patterns relate to molecular markers in cancer patients. The LIFELOG study aims to address this knowledge gap by integrating continuous PA monitoring with comprehensive molecular profiling in post-surgical cancer patients, ultimately contributing to more personalized treatment strategies that incorporate PA recommendations based on molecular profiles.

## The SCRUM-MONSTAR LIFELOG study

### Study design

The SCRUM-MONSTAR LIFELOG study (Institutional Review Board number: 2024-111) is a prospective observational sub-study of MONSTAR-SCREEN-3 platform trial (UMIN000053975). MONSTAR-SCREEN-3 is a Japanese nationwide initiative employing artificial intelligence-driven multi-omics analyses in cancer patients, including tumor-informed WGS-based MRD detection, ST, proteomics, germline analysis, and microbiome analyses [27, 28]. The study incorporates electronic patient-reported outcomes (ePROs) using validated instruments including the European Organisation for Research and Treatment of Cancer Quality of Life Questionnaire Core 30 (EORTC QLQ-C30) and the 5-level EuroQol 5-Dimension (EQ-5D-5L) questionnaires. In addition, the platform systematically collects and digitizes pathological specimens and radiological imaging data, enabling comprehensive digital pathology analysis and radiomics evaluation. This multi-modal approach facilitates an integrated assessment of PA’s impact on cancer biology and patient outcomes.

### Study population

Patients who have undergone curative surgery for colorectal cancer, renal cell carcinoma, urothelial carcinoma, endometrial cancer, or gastric cancer (cardia) will be enrolled. These cancer types were selected based on established evidence linking PA to outcomes in these malignancies [2].

Inclusion criteria

- Enrollment in MONSTAR-SCREEN-3 trial,
- Achievement of R0/R1 resection through curative surgery,
- No evidence of disease recurrence,
- Eastern Cooperative Oncology Group (ECOG) Performance Status of 0–2,
- Completion of baseline and 1-month post-surgical blood sampling for MRD analysis,
- Written informed consent.

Exclusion criteria

- Unwillingness to return the wearable device,
- Deemed unsuitable for participation by treating physician.

### Sample size and study phases

The study consists of a feasibility phase and an expansion phase. The feasibility phase will enroll 20 patients to evaluate:

- MRD positivity and clearance rates,
- Wearable device compliance (defined as ≥ 10 h/day for ≥ 3 days/week),
- Data collection completeness,
- Initial correlation between PA metrics and MRD status,
- Effect size estimation.

The feasibility phase has already commenced and is progressing successfully, with preliminary data indicating high device compliance rates and robust data collection. These encouraging results have confirmed the feasibility of the full study protocol, allowing us to proceed to the main study phase. We are currently awaiting the MRD analysis results, which will be used to fine-tune the final enrollment number for the expansion phase. Based on these feasibility phase metrics, particularly MRD detection rates and observed correlation with PA, the final sample size for the expansion phase will be calculated using standard statistical parameters (one-sided alpha = 0.1, power = 0.80). While currently planned for 150 additional patients, this number may be adjusted based on the feasibility results. Statistical significance levels may also be modified if needed based on observed effect sizes.

### Study procedures

Participants will wear the mSafety™ wrist device [29] continuously except during bathing and sleeping. The device collects triaxial acceleration data (32 Hz sampling, ± 8.0G range), triaxial gyroscope data (32 Hz, ± 500 degrees/second), and photoplethysmography signals from a built-in PPG sensor. Raw sensor data are encrypted and automatically transmitted via LTE-M to a secure cloud server, allowing researchers direct access to unprocessed data for analysis. From this raw sensor data, several specific physical activity parameters are expected to be derived. For overall activity assessment, the device enables calculation of validated metrics such as Euclidean Norm Minus One (ENMO) [30] for overall activity volume quantification, total daily activity counts, and estimation of active minutes at different intensity levels. Regarding activity patterns, the data allow identification of VILPA [1] episodes, continuous activity bouts, and daily activity distribution patterns. The PPG sensor provides physiological response data, including heart rate patterns during activities and heart rate variability indices, which serve as indicators of autonomic function. In addition, the continuous monitoring nature of the device offers contextual information such as estimates of sedentary time and wear time validation.

**Fig. 1:**
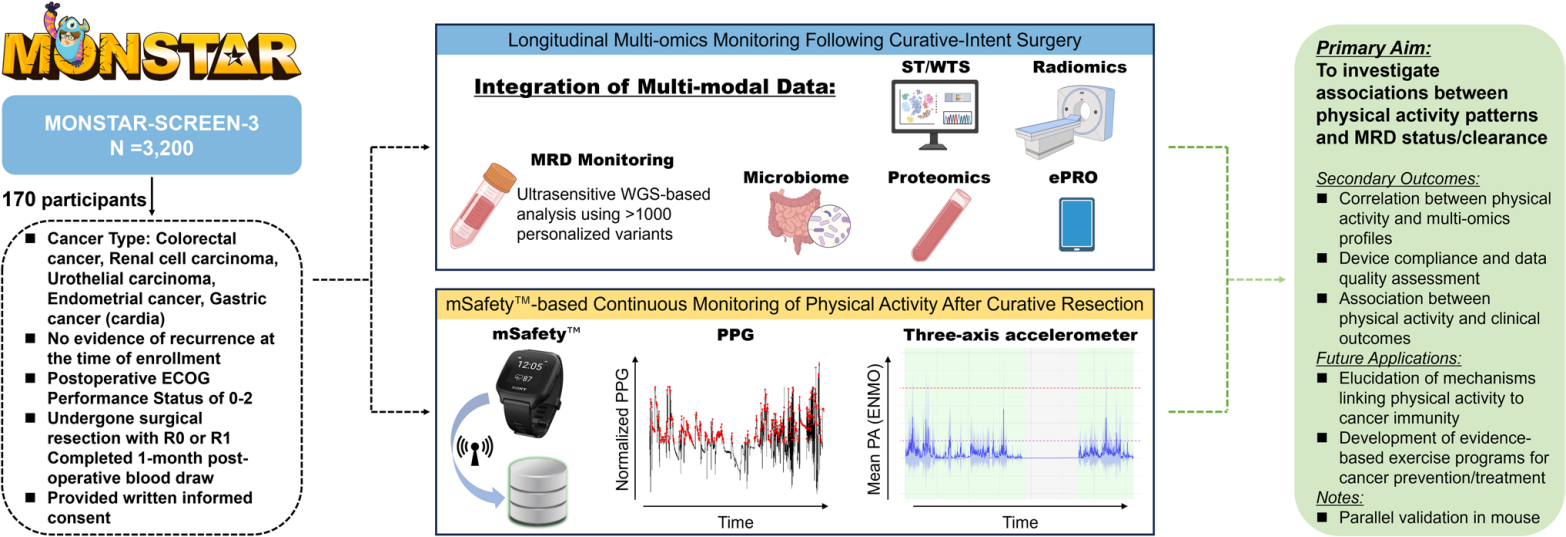
LIFELOG study: multi-modal analysis integrating physical activity monitoring and molecular residual disease detection for post-surgical cancer patients. Note: Illustration created with BioRender.com, accessed on 25 January 2025. *ECOG* Eastern Cooperative Oncology Group, *ENMO* Euclidean Norm Minus One, *ePRO* electronic patient-reported outcome, *MRD* molecular residual disease, *PA* physical activity, *PPG* PHOTOPLETHYSMOGRAPHY, *R0/R1* resection margins (R0: no residual tumor, R1: microscopic residual tumor), *ST/WTS* spatial transcriptome/whole transcriptome sequencing, *WGS* whole-genome sequencing

The study leverages MONSTAR-SCREEN-3’s comprehensive molecular profiling infrastructure, including WGS-based ctDNA analysis for MRD monitoring, blood proteomics, spatial transcriptome of tumor tissue, and microbiome analysis, alongside AI-enhanced digital pathology and radiomics. Particularly relevant to understanding exercise-induced effects on immune function and inflammation, the plasma proteomics analyses will enable quantification of inflammatory markers, immune-related cytokines, myokines, and metabolic regulators. This direct measurement of the systemic inflammatory and immune environment in relation to physical activity patterns may help elucidate the biological mechanisms through which exercise influences MRD clearance. The spatial transcriptomics analysis of tumor tissue will provide complementary data on the tumor immune microenvironment, potentially revealing exercise-associated changes in immune cell infiltration and inflammatory signaling pathways within the tumor. This integrated approach enables correlation between objectively measured PA patterns and molecular/clinical outcomes. Figure 1 provides an overview of the study design and data collection workflow.

### Data management

PA data will be encrypted and transmitted to a cloud server before secure transfer to the National Cancer Center Hospital East. All data will be managed in accordance with institutional policies and relevant privacy regulations.

### Outcome measures

The primary outcome is the association between PA metrics (including but not limited to total activity volume, frequency and total duration of VILPA) and MRD status/clearance. Secondary outcomes include device compliance rate, correlation between PA patterns and molecular profiles, and association between PA and clinical outcomes. These clinical outcomes include recurrence-free survival (RFS), overall survival (OS), disease-specific survival, time to recurrence, treatment-related adverse events, post-surgical recovery metrics (length of hospital stay, time to return to baseline activities), and frequency of unplanned healthcare utilization. Patient-reported PA and quality of life data will be collected through ePRO questionnaires and analyzed for correlation with objective activity measurements from the mSafety™ device. In addition, radiological images will be digitized and analyzed using artificial intelligence for comprehensive radiomics evaluation. This analysis will be performed on both tumor regions (assessing characteristics such as texture, heterogeneity, and morphological features) and skeletal muscles (evaluating muscle mass, density, and composition). This dual-focused radiomics approach aims to investigate potential associations between PA patterns and changes in both tumor characteristics and body composition metrics over time.

### Statistical analysis

MRD positivity is defined as detectable ctDNA at 1-month post-surgery, with clearance defined as conversion from positive to negative status during follow-up. Associations between PA metrics and molecular markers will be analyzed using appropriate statistical methods while controlling for relevant clinical variables, such as age, sex, cancer type, stage, and treatment regimen. Detailed statistical analysis plans, including specific methods and statistical models, will be developed based on Phase 1 feasibility data and finalized prior to the analysis of the full cohort data.

## Discussion

The LIFELOG study represents a novel approach to understanding the relationship between PA and cancer outcomes through continuous high-fidelity activity monitoring integrated with molecular profiling. The successful implementation of the feasibility phase has already demonstrated the practical viability of our study design, particularly regarding participant adherence to the wearable device protocol and the quality of collected data. This early success provides a strong foundation for the expansion phase, although final adjustments to sample size will be guided by the pending MRD analysis results. The mSafety™ device’s 32 Hz sampling rate for triaxial acceleration combined with PPG data enables comprehensive characterization of PA patterns. While acceleration data capture intensity, frequency, and duration of movement, the integration of PPG signals provides complementary physiological data such as heart rate variability and peripheral perfusion changes during activity. This multi-modal activity monitoring may offer more precise insights into exercise intensity and physiological responses than acceleration data alone [29].

The study’s integration within MONSTAR-SCREEN-3’s comprehensive molecular profiling infrastructure [27, 28] provides unprecedented ability to correlate detailed activity measurements with WGS-based MRD dynamics, changes in immune cell populations and spatial organization via ST analysis, systemic adaptations through proteomics and microbiome analyses, and tumor evolution through longitudinal molecular profiling. These multi-modal analyses may reveal specific biological mechanisms linking PA characteristics to anti-tumor responses.

To validate findings from the human cohort and establish mechanistic proof-of-concept, parallel studies in mouse models are planned. These experiments will examine how controlled exercise interventions affect tumor growth, immune cell infiltration, and molecular profiles in cancer-bearing mice. The animal studies can systematically evaluate different exercise parameters and timing relative to tumor progression, providing causal evidence to complement the observational human data. Integration of findings from both human monitoring and mouse experiments may help identify the most crucial aspects of PA for cancer outcomes.

Several practical considerations warrant attention. The initial feasibility phase will assess compliance with continuous monitoring and data quality. Managing the high-dimensional data from both activity monitoring and multi-omics profiling requires robust data infrastructure and analytics. The observational design of the human cohort limits causal inference about activity effects, highlighting the importance of the complementary mouse studies.

By elucidating the relationships between objectively measured PA patterns and molecular cancer dynamics in both humans and mice, this study may help optimize activity recommendations for cancer prevention and treatment. Understanding which characteristics of PA most strongly influence cancer outcomes could guide development of more precise, evidence-based exercise prescriptions that consider intensity, timing, and physiological responses rather than just total duration. These insights may ultimately contribute to more personalized and effective integration of PA into comprehensive cancer care.

## Data Availability

To protect patient privacy and confidentiality, the clinical and activity tracking data from this study are not publicly available. However, de-identified data may be available from the corresponding author upon reasonable request and with appropriate approval from the study steering committee. Any data sharing requests will be reviewed to verify whether the request is subject to any intellectual property or confidentiality obligations.

## References

[1] Stamatakis E, Ahmadi MN, Gill JMR et al (2022) Association of wearable device-measured vigorous intermittent lifestyle physical activity with mortality. Nat Med 28:2521–2529. 10.1038/s41591-022-02100-x 36482104 10.1038/s41591-022-02100-x PMC9800274

[2] Moore SC, Lee IM, Weiderpass E et al (2016) Association of leisure-time physical activity with risk of 26 types of cancer in 1.44 million adults. JAMA Intern Med 176:816–825. 10.1001/jamainternmed.2016.1548 27183032 10.1001/jamainternmed.2016.1548 PMC5812009

[3] Friedenreich CM, Stone CR, Cheung WY et al (2019) Physical activity and mortality in cancer survivors: a systematic review and meta-analysis. JNCI Cancer Spectr 4:pkz080. 10.1093/jncics/pkz080 32337494 10.1093/jncics/pkz080 PMC7050161

[4] McTiernan A, Friedenreich CM, Katzmarzyk PT et al (2019) Physical activity in cancer prevention and survival: a systematic review. Med Sci Sports Exerc 51:1252–1261. 10.1249/MSS.0000000000001937 31095082 10.1249/MSS.0000000000001937 PMC6527123

[5] Feng Y, Feng X, Wan R et al (2024) Impact of exercise on cancer: mechanistic perspectives and new insights. Front Immunol 15:1474770. 10.3389/fimmu.2024.1474770 39346906 10.3389/fimmu.2024.1474770 PMC11427289

[6] Fiuza-Luces C, Valenzuela PL, Gálvez BG et al (2024) The effect of physical exercise on anticancer immunity. Nat Rev Immunol 24:282–293. 10.1038/s41577-023-00939-w 37794239 10.1038/s41577-023-00943-0

[7] Yang L, Morielli AR, Heer E et al (2021) Effects of exercise on cancer treatment efficacy: a systematic review of preclinical and clinical studies. Cancer Res 81:4889–4895. 10.1158/0008-5472.CAN-21-1258 34215623 10.1158/0008-5472.CAN-21-1258 PMC9397632

[8] Deng N, Reyes-Uribe L, Fahrmann JF et al (2023) Exercise training reduces the inflammatory response and promotes intestinal mucosa-associated immunity in lynch syndrome. Clin Cancer Res 29:4361–4372. 10.1158/1078-0432.CCR-23-0088 37724990 10.1158/1078-0432.CCR-23-0088 PMC10618653

[9] Kyu HH, Bachman VF, Alexander LT et al (2016) Physical activity and risk of breast cancer, colon cancer, diabetes, ischemic heart disease, and ischemic stroke events: systematic review and dose-response meta-analysis for the global burden of disease study 2013. BMJ. 10.1136/bmj.i3857 27510511 10.1136/bmj.i3857 PMC4979358

[10] Friedenreich CM, Neilson HK, Lynch BM (2010) State of the epidemiological evidence on physical activity and cancer prevention. Eur J Cancer 46:2593–2604. 10.1016/j.ejca.2010.07.028 20843488 10.1016/j.ejca.2010.07.028

[11] Zhou Y, Jia N, Ding M et al (2022) Effects of exercise on inflammatory factors and IGF system in breast cancer survivors: a meta-analysis. BMC Womens Health 22:507. 10.1186/s12905-022-02058-5 36482346 10.1186/s12905-022-02058-5 PMC9730577

[12] Khandekar MJ, Cohen P, Spiegelman BM (2011) Molecular mechanisms of cancer development in obesity. Nat Rev Cancer 11:886–895. 10.1038/nrc3174 22113164 10.1038/nrc3174

[13] Beavers KM, Brinkley TE, Nicklas BJ (2010) Effect of exercise training on chronic inflammation. Clin Chim Acta 411:785–793. 10.1016/j.cca.2010.02.069 20188719 10.1016/j.cca.2010.02.069 PMC3629815

[14] Radak Z, Zhao Z, Koltai E et al (2013) Oxygen consumption and usage during physical exercise: the balance between oxidative stress and ROS-dependent adaptive signaling. Antioxid Redox Signal 18:1208–1246. 10.1089/ars.2011.4498 22978553 10.1089/ars.2011.4498 PMC3579386

[15] Hojman P, Gehl J, Christensen JF et al (2018) Molecular mechanisms linking exercise to cancer prevention and treatment. Cell Metab 27:10–21. 10.1016/j.cmet.2017.09.015 29056514 10.1016/j.cmet.2017.09.015

[16] Pedersen L, Idorn M, Olofsson GH et al (2016) Voluntary running suppresses tumor growth through epinephrine- and IL-6-dependent NK cell mobilization and redistribution. Cell Metab 23:554–562. 10.1016/j.cmet.2016.01.011 26895752 10.1016/j.cmet.2016.01.011

[17] Santa Mina D, Clarke H, Ritvo P et al (2014) Effect of total-body prehabilitation on postoperative outcomes: a systematic review and meta-analysis. Physiotherapy 100:196–207. 10.1016/j.physio.2013.08.008 24439570 10.1016/j.physio.2013.08.008

[18] Barberan-Garcia A, Ubré M, Roca J et al (2018) Personalised prehabilitation in high-risk patients undergoing elective major abdominal surgery: a randomized blinded controlled trial. Ann Surg 267:50–56. 10.1097/SLA.0000000000002293 28489682 10.1097/SLA.0000000000002293

[19] van Vulpen JK, Peeters PH, Velthuis MJ et al (2016) Effects of physical exercise during adjuvant breast cancer treatment on physical and psychosocial dimensions of cancer-related fatigue: a meta-analysis. Maturitas 85:104–111. 10.1016/j.maturitas.2015.12.007 26857888 10.1016/j.maturitas.2015.12.007

[20] Koelwyn GJ, Quail DF, Zhang X et al (2017) Exercise-dependent regulation of the tumour microenvironment. Nat Rev Cancer 17:620–632. 10.1038/nrc.2017.78 28943640 10.1038/nrc.2017.78

[21] Schumacher O, Galvão DA, Taaffe DR et al (2021) Exercise modulation of tumour perfusion and hypoxia to improve radiotherapy response in prostate cancer. Prostate Cancer Prostatic Dis 24:1–14. 10.1038/s41391-020-0245-z 32632128 10.1038/s41391-020-0245-z PMC8012204

[22] Liu J, Liu W, Wan Y et al (2024) Crosstalk between exercise and immunotherapy: current understanding and future directions. Research (Wash D C) 7:0360. 10.34133/research.0360 38665847 10.34133/research.0360 PMC11045263

[23] Mustian KM, Alfano CM, Heckler C et al (2017) Comparison of pharmaceutical, psychological, and exercise treatments for cancer-related fatigue: a meta-analysis. JAMA Oncol 3:961–968. 10.1001/jamaoncol.2016.6914 28253393 10.1001/jamaoncol.2016.6914 PMC5557289

[24] Craft LL, Vaniterson EH, Helenowski IB et al (2012) Exercise effects on depressive symptoms in cancer survivors: a systematic review and meta-analysis. Cancer Epidemiol Biomarkers Prev 21:3–19. 10.1158/1055-9965.EPI-11-0634 22068286 10.1158/1055-9965.EPI-11-0634 PMC3253916

[25] Kotani D, Oki E, Nakamura Y et al (2023) Molecular residual disease and efficacy of adjuvant chemotherapy in patients with colorectal cancer. Nat Med 29:127–134. 10.1038/s41591-022-02133-2 36646802 10.1038/s41591-022-02115-4 PMC9873552

[26] Kobayashi S, Nakamura Y, Hashimoto T et al (2025) Japan society of clinical oncology position paper on appropriate clinical use of molecular residual disease (MRD) testing. BMC Cancer 2:605–654 10.1007/s10147-024-02683-0 PMC11946966 39920551

[27] Hashimoto T, Nakamura Y, Fujisawa T et al (2024) The SCRUM-MONSTAR cancer-omics ecosystem: striving for a quantum leap in precision medicine. Cancer Discov 14:2243–2261. 10.1158/2159-8290.CD-23-0980 39023403 10.1158/2159-8290.CD-24-0206 PMC11528206

[28] Hashimoto T, Nakamura Y, Oki E et al (2024) Bridging horizons beyond CIRCULATE-Japan: a new paradigm in molecular residual disease detection via whole genome sequencing-based circulating tumor DNA assay. Int J Clin Oncol 29:495–511. 10.1007/s10147-024-02541-1 38551727 10.1007/s10147-024-02493-4 PMC11043144

[29] Hayano J, Adachi M, Sasaki F et al (2024) Quantitative detection of sleep apnea in adults using inertial measurement unit embedded in wristwatch wearable devices. Sci Rep 14:4050. 10.1038/s41598-024-51651-1 38374225 10.1038/s41598-024-54817-z PMC10876631

[30] Bai J, Di C, Xiao L et al (2016) An activity index for raw accelerometry data and its comparison with other activity metrics. PLoS ONE. 10.1371/journal.pone.0160644 27513333 10.1371/journal.pone.0160644 PMC4981309

